# Infographic: residual intraretinal edema after 25-gauge vitrectomy and macular pucker removal: Is intraoperative sustained-release dexamethasone a real treatment option?

**DOI:** 10.1038/s41433-021-01533-x

**Published:** 2021-05-10

**Authors:** M. Al-Zubaidy, A. Ghareeb, I. Mostafa, A. Mehta, D. Murphy, S. Sadiq, A. Song, N. Tzoumas, D. H. Steel

**Affiliations:** Institute of Biosciences, Newcastle University, Newcastle upon Tyne, UK

**Keywords:** Retinal diseases, Diagnosis

**Reference to original study:**

Guidi G, Casini G, Ripandelli G, Piaggi P, Lucche FD, Sartini M, Loiudice P, Nasini F, Stripe M, Lazzeri S. Residual intraretinal edema after 25-gauge vitrectomy and macular pucker removal: Is intraoperative sustained-release dexamethasone a real treatment option? Retina. 2018;38:993–9. 10.1097/IAE.0000000000001627.

**Fig. 1:**
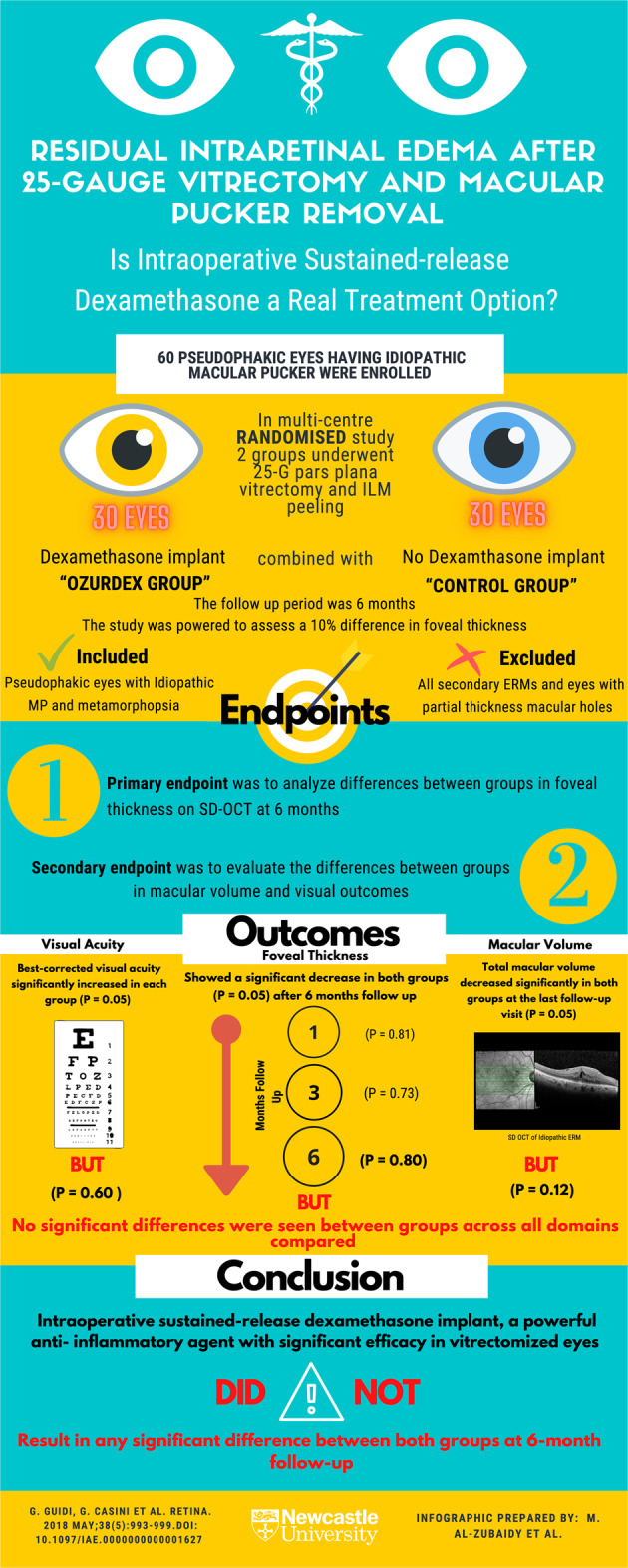
An infographic looking at the efficacy of the use of intraoperative sustained-release dexamethasone in patients who underwent 25-gauge vitrectomy and macular pucker removal. This randomised controlled trial aimed to investigate the efficacy and safety of intraoperative slow-release dexamethasone implant in vitrectomy plus OZRUDEX implant vs. vitrectomy without OZRUDEX implant for the treatment of idiopathic macular pucker. It found that intraoperative sustained-release dexamethasone implant, a powerful anti-inflammatory agent with significant efficacy in vitrectomized eyes, did not result in a significant change in macular thickness and volume compared with the vitrectomy alone without dexamethasone implant at 6-month follow-up. ERM epiretinal membrane, MP macular pucker.

